# Global studies in social determinants of health in people with Alzheimer´s disease

**DOI:** 10.1002/alz70860_097653

**Published:** 2025-12-23

**Authors:** Maryenela Illanes‐Manrique, Pilar Giron‐Davila, Larry D Adams, Sheila Castro‐Suarez, Julia Rios‐Pinto, Pedro R Mena, Elison Sarapura‐Castro, Farid Rajabli, Ivan Cornejo‐Herrera, Katalina F. McInerney, Michael L Cuccaro, Jeffery M Vance, Angel Medina‐Colque, Koni Mejia‐Rojas, Edward Ochoa‐Valle, Serggio Lanata, Aldo Vences‐Balta, Eden R. Martin, Giuseppe Tosto, Margaret Pericak‐Vance, Mario Cornejo‐Olivas

**Affiliations:** ^1^ Neurogenetics Research Center, Instituto Nacional de Ciencias Neurológicas, Lima, Peru; ^2^ Atlantic Fellow for Equity in Brain Health at Global Brain Health Institute (GBHI), San Francisco, CA, USA; ^3^ Neurogenetics Working Group, Universidad Cientifica del Sur, Lima, Peru; ^4^ Dr. John T. Macdonald Foundation Department of Human Genetics, University of Miami Miller School of Medicine, Miami, FL, USA; ^5^ John P. Hussman Institute for Human Genomics, University of Miami Miller School of Medicine, Miami, FL, USA; ^6^ CBI en Demencias y Enfermedades Desmielinizantes del Sistema Nervioso, Instituto Nacional de Ciencias Neurológicas, Lima, Peru; ^7^ Universidad Peruana Los Andes, Huancayo, Peru; ^8^ University of Miami Miller School of Medicine, Miami, FL, USA; ^9^ Hospital Hipolito de Tacna, Tacna, Peru; ^10^ Dirección Regional de Salud de Puno, Puno, Peru; ^11^ EDMECON Continuing Medical Education, Lima, Peru; ^12^ Daniel Alcides Carrion National Hospital, Callao, Peru; ^13^ Hospital Regional de Cusco, Cusco, Peru; ^14^ Memory and Aging Center, UCSF Weill Institute for Neurosciences, University of California, San Francisco, San Francisco, CA, USA; ^15^ Centro Medico Regional Piura (CEMERP), Piura, Peru; ^16^ Columbia University, New York, NY, USA

## Abstract

**Background:**

Social, behavioral, and environmental determinants of health (SDOH), such as access to and quality of education and healthcare, and socioeconomic factors, influence health outcomes, including dementia. We aim to identify the main SDOH associated with Alzheimer's disease (AD) by comparing AD cases to cognitively unimpaired (CU) individuals in the Peruvian population recruited in the Peruvian Alzheimer´s Disease Initiative (PeADI) and Global LAtino Sequencing Study for Alzheimer's Disease (GLASS‐AD) studies. These studies are critical to advance our understanding to solve AD for everyone.

**Method:**

The Social Determinants of Brain Health Questionnaire (SDOBH‐Q) underwent expert review by local and international neurologists/psychiatrists and local linguistic adaptation. We administer the revised questionnaire to over 200 AD cases and 200 CU individuals from ongoing genetic studies of AD (PeADI and GLASS) in Peru. This includes participants from three predominantly rural regions in the Andean highlands (Huancayo, Cusco, Puno) and four coastal urban regions (Lima, Callao, Tacna and Piura). The association between identified SDOH and AD status will be analyzed using multivariable regressions, adjusted for age, gender, and population substructure.

**Result:**

The revised SDOBH‐Q questionnaire consists of 15 sections and 79 questions. The pilot assessment was completed by 28 CU volunteers: 17 from Lima and 11 from Huancayo. Preliminary data from the first 170 participants have been collected across regions from the coast and the Andean highlands of Peru, showing differences between AD and CU regarding neighborhood, education, healthcare access, and economic conditions.

**Conclusion:**

Specific SDOH, such as neighborhood, education, healthcare access, economic conditions, and environmental factors, may increase the risk of AD in older Peruvian individuals. Gaining a complete understanding of how genetic and environmental factors interact in the context of AD and related dementias globally is critical to our understanding of the disease and to the development of future preventions and treatments.

